# Advanced MRI for carotid plaque imaging

**DOI:** 10.1007/s10554-015-0743-6

**Published:** 2015-08-21

**Authors:** Navneet Singh, Alan R. Moody, Idan Roifman, David A. Bluemke, Anna E. H. Zavodni

**Affiliations:** Department of Medical Imaging, Sunnybrook Health Sciences Centre, University of Toronto, 2075 Bayview Avenue, Room AG56b, Toronto, ON M4N 3M5 Canada; Division of Cardiology, Department of Internal Medicine, Sunnybrook Health Sciences Centre, University of Toronto, Toronto, Canada; Radiology and Imaging Sciences, Clinical Center, National Institutes of Health, Bethesda, MD USA

**Keywords:** Atherosclerosis, Imaging, Carotid, MRI, Cardiovascular risk

## Abstract

Atherosclerosis is the ubiquitous underling pathological process that manifests in heart attack and stroke, cumulating in the death of one in three North American adults. High-resolution magnetic resonance imaging (MRI) is able to delineate atherosclerotic plaque components and total plaque burden within the carotid arteries. Using dedicated hardware, high resolution images can be obtained. Combining pre- and post-contrast T1, T2, proton-density, and magnetization-prepared rapid acquisition gradient echo weighted fat-saturation imaging, plaque components can be defined. Post-processing software allows for semi- and fully automated quantitative analysis. Imaging correlation with surgical specimens suggests that this technique accurately differentiates plaque features. Total plaque burden and specific plaque components such as a thin fibrous cap, large fatty or necrotic core and intraplaque hemorrhage are accepted markers of neuroischemic events. Given the systemic nature of atherosclerosis, emerging science suggests that the presence of carotid plaque is also an indicator of coronary artery plaque burden, although the preliminary data primarily involves patients with stable coronary disease. While the availability and cost-effectiveness of MRI will ultimately be important determinants of whether carotid MRI is adopted clinically in cardiovascular risk assessment, the high accuracy and reliability of this technique suggests that it has potential as an imaging biomarker of future risk.

## Introduction

Cardiovascular disease claims the lives of at least one in three North American adults, with atherosclerosis as the leading cause of cardiovascular related-mortality and morbidity [1]. While traditional cardiovascular risk factors obtained from the patient’s history, physical exam and biochemical markers may be used to predict coronary heart disease [2], composite scoring systems calibrated for cardiac disease, such as the Framingham risk score model, do not adequately predict incident stroke [3]. These traditional scores can also underestimate the risk of cardiovascular disease in women [4] and socioeconomically deprived individuals [5]. These risk models do not adequately account for all of the inherited, anatomical and environmental variables contributing to cardiovascular events [6]. Direct atherosclerotic imaging can provide insight into the total plaque burden, composition and stability. Carotid MRI has proven to be a useful adjunct in reclassifying patients at risk [7].

The carotid bifurcation is a region of unique vulnerability. The branching point is the focus of elevated shear-stress. This elevated tension occurs at the junction between the internal carotid artery, supplying the low-pressure cerebral circulation, and the external carotid branch, providing blood to the high resistance facial muscles [8]. This vulnerable region is well suited for imaging evaluation and provides an ideal surrogate for other vascular beds. Superficially located, the carotid arteries are easily palpated, allowing for the precise positioning of surface coils. Compared to the coronary vasculature, the carotid arteries are large and relatively immobile, reducing motion artifact. Thus, since the carotid arteries are susceptible to early atherosclerotic damage, superficially situated and essentially stationary, these vessels are optimally suited for imaging study.

## Comparison to other techniques

Carotid MRI has many advantages over other imaging techniques. While ultrasound is a widely available method that is commonly used for screening, its spatial, temporal and contrast resolution is limited, reducing its accuracy for evaluating carotid stenosis [9] and plaque components [10] relative to MRI. Computed tomography (CT) has high spatial resolution but involves ionizing radiation and the imaging of heavily calcified lesions can overestimate the burden of disease [11]. Positron emission tomography (PET) is valuable for the characterization of plaque inflammation but is unable to accurately depict other plaque features [12, 13]. Thus of all of the commonly utilized noninvasive clinical imaging modalities, MRI is the most accurate and versatile.

## MR hardware

Optimal vascular imaging requires high-field magnetic resonance systems which may be coupled with dedicated surface coils. Several studies comparing T1-, T2-, and proton density-weighted black-blood techniques at 1.5- and 3-T have observed significant improvements in the signal-to-noise (SNR) and contrast-to-noise ratios and the overall image quality using the higher field strength system [14–16]. Further improvements to image quality can be achieved through the use of dedicated surface coils by boosting the SNR and minimizing the propagation of flow artifacts [17, 18].

**Fig. 1:**
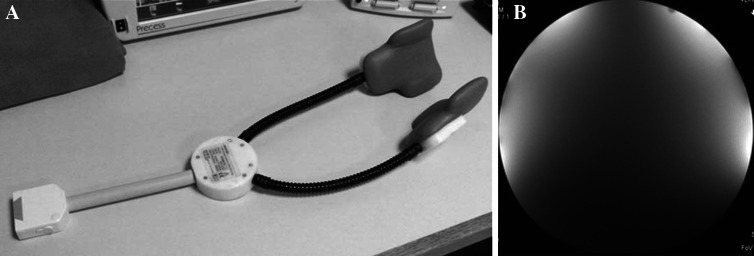
Dedicated surface coils provide improved signal-to-noise ratio for superficial structures (**a**). When these coils are applied to a cylindrical water phantom, measuring 6 cm in diameter, the drop-off in signal intensity on the T1-weighted images provides a visual demonstration of the penetration depth of the coil (**b**)

**Fig. 2:**
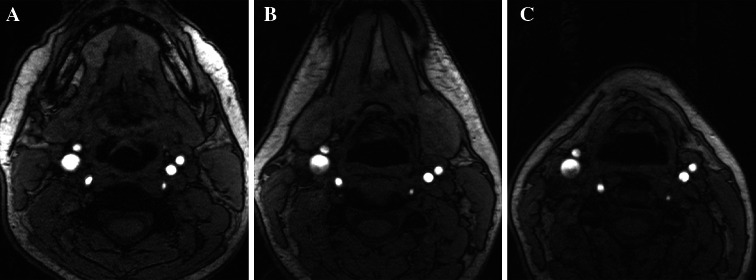
Patient positioning can significantly alter the depth of the carotid arteries relative to overlying muscle, grandular tissue and skin as demonstrated by these time-of-flight images obtained during the same imaging session with the patient’s neck flexed (**a**), in neutral position (**b**) and extended (**c**)

As illustrated in Figs. 1 and 2, surface coils require careful positioning. Figure 1 depicts the coils positioned over a water phantom and demonstrates a sharp drop in signal with depth. The position of the bifurcation can vary significantly with neck motion. As illustrated in Fig. 2, flexion can superimpose the jaw bone and submandibular soft tissues over the carotid bifurcation, thus increasing the depth of the carotid bulb and reducing the efficacy of the surface coils. Therefore, image quality is dependent on both hardware and technical expertise in the use of this equipment.

## Plaque characterization

First described by Glagov et al. [19], the morphological changes of atherogenesis begin with an outward expansion the vessel. Demonstrated initial on pathological specimens and later with MRI [20], the artery undergoes compensatory dilation with eccentric remodeling before further plaque deposition causes luminal encroachment.

**Fig. 3:**
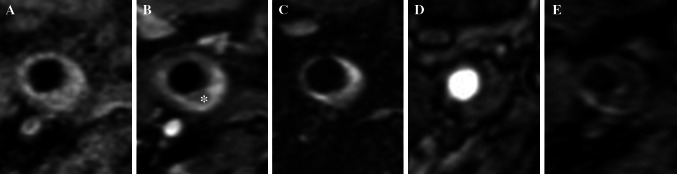
MRI allows depiction of several atherosclerotic components including lipid core (*asterisk*). Signal hypointensity (*asterisk*) indicates the lipid core of an eccentric atherosclerotic plaque with luminal preservation on fat-saturation T1-weighted imaging pre- (**a**), and post-contrast (**b**). Multi-contrast images usually also include T2 fat saturation (**c**), time-of-flight (**d**), and MPRAGE (**e**)

**Fig. 4:**
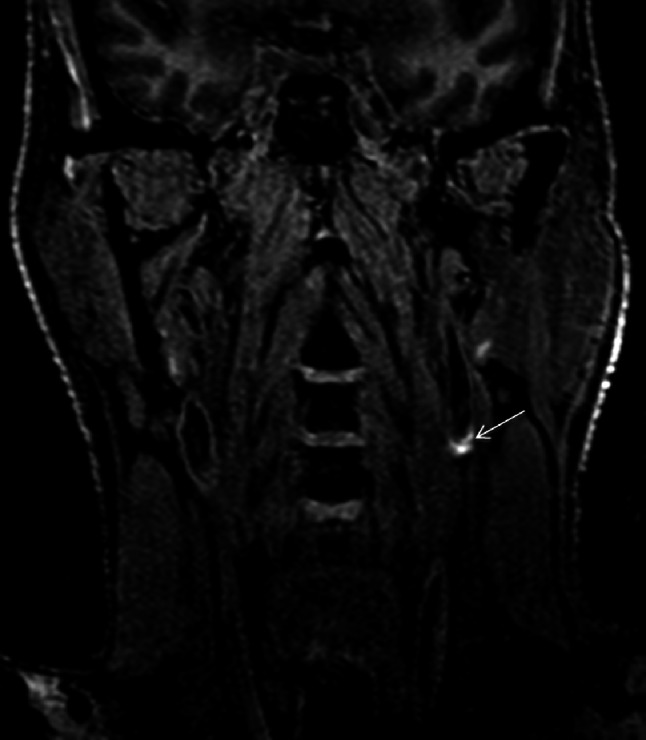
Coronal T1-weighted IR 3D FFE image depicting a hyperintensity in the left carotid artery indicating intraplaque hemorrhage. Hyperintense signal in the carotid wall >150 % of the adjacent sternocleidomastoid muscle on this sequence accurately and reliably depicts intraplaque hemorrhage

Identifying plaque components, including the presence or absence of a lipid core, fibrous cap, fibrous tissue components and calcification can be achieved by varying the image acquisition parameters (see Fig. 3). Flow-suppressed T1-weighted studies before and after contrast, T2 and proton-density weighted imaging are routinely used in carotid assessment [21–23]. T1-weighted, fat and flow suppressed sequences are best to evaluate intra-plaque hemorrhage, exploiting methemoglobin induced T1-shortening (Fig. 4) [24–26]. More recent publications suggest that the acquisition of various contrast weighting can be minimized to pre- and post-contrast T1-weighted, fat and flow suppressed and time-of-flight imaging, eliminating the time necessary for the proton-density and T2-weighted imaging acquisition, while maintaining the ability to quantify plaque morphology and identify the most clinically relevant composition features including the presence of the lipid-rich necrotic core and a thin fibrous cap [27, 28]. Table 1 provides an overview of the typical patterns of imaging signal intensity associated with the various components of atherosclerotic plaque [21, 22, 29]. The parameters described in Table 1 been studied extensively and correlated with histopathology [29–32].

**Table 1:** Contrast of MRI plaque components

	T1 pre	T1 post	T2	PD	TOF
Lipid core	Iso/high	Low		Low	Low
Fibrous cap	Iso	Iso	Mixed	Mixed	Low
Fibrous tissue	Iso/high	v. high	Iso/high	Iso/high	Low
Hemorrhage	v. high		Variable	Variable	Variable
Calcification	Low	Low	Low	Low	Low

Imaging-histopathological correlation of atherosclerotic plaque has demonstrated patterns of fat-saturated T1 pre- and post-contrast, T2 and proton-density and time-of-flight (TOF) signal intensity that differentiates lipid core, fibrous cap and tissue components, hemorrhage and calcification [21, 22, 29]

*Iso* isointense to skeletal muscle, *v.* very

Depending upon the imaging parameters, cardiac-gating may no longer be necessary. In the past, single-slice cardiac-triggered black-blood acquisitions have been obtained, effectively suppressing flow artifacts around the carotid bifurcation [33], however, these gated techniques prolong the total examination time, potentially incurring greater study costs and compromising patient comfort. More recently, inflow and outflow saturation techniques have been incorporated into black-blood techniques, allowing non-gated sequences to be acquired without impairing image quality [34].

Contrast agents can enhance the characterization of the arterial lumen and carotid wall. Contrast-enhanced MR angiography improves the accuracy of high grade stenosis evaluation over 3D time-of-flight angiography [35]. Delayed enhancement imaging improves the visualization of plaque components, and enhancing regions strongly correlate with regions of neovascularity and inflammation on histology. Inflammation is depicted even better by ultrasmall superparamagnetic iron oxide (USPIO) particles. This material is phagocytized by macrophages and its subsequent accumulation within inflammatory cells can be detected as signal drop-out on T2-weighted sequences [36]. These particles are used to distinguish inflammatory components of symptomatic and asymptomatic plaque [37].

Another important aspect of carotid vessel characterization is the detection of intraluminal thrombus. Plaque rupture exposes the circulating blood to thrombogenic material, subsequently resulting in thrombus formation that may occlude the artery or embolize distally. In the setting of acute stroke, susceptibility-weighted imaging has been used to demonstrate intra-arterial thrombus [38], demonstrating improved sensitivity for the detection of intraluminal disease compared to time-of-flight angiography [39] and contrast-enhanced imaging [40].

## Flow measurements

The inspection of pathology specimens has demonstrated that atherosclerotic plaque predominately develops adjacent to the bends and major branches within any particular arterial network [41]. These findings suggest that a disruption of geometry alters flow dynamics and contributes to the induction of atherosclerotic plaque [42]. MRI allows for the comprehensive characterization of carotid bulb geometry, including luminal diameter, wall thickness and volume and vascular tortuosity. The bifurcation geometry independently predicts wall thickening [43]. Within the carotid bifurcation, the admixture of low-pressure internal- and high pressure external-carotid circulation creates a region of non-laminar flow and elevated shear-stress, assumed to potentiate atherogenesis. Wall shear-stress has been estimated through the combination of MRI phase contrast imaging and computational fluid dynamic techniques that incorporate information regarding vessel geometry and measurements of flow [44].

MRI can be further used to assess complex flow patterns. Early phantom and patient studies [45] have demonstrated the efficacy of differing sequences in depicting flow under various conditions. Steady-state free precession imaging is a balanced technique that optimally depicts the lumen under no-flow and slow flow conditions. Time-of-flight imaging produces good opacity provided there is moderate blood velocity and not excessive intravoxel dephasing from fast or in-plane flow. As described in the section above, black-blood fast- or turbo-spin echo techniques best eliminate artifact with inflow and outflow suppression techniques and perform well with higher flow velocities.

## Post-processing

**Fig. 5:**
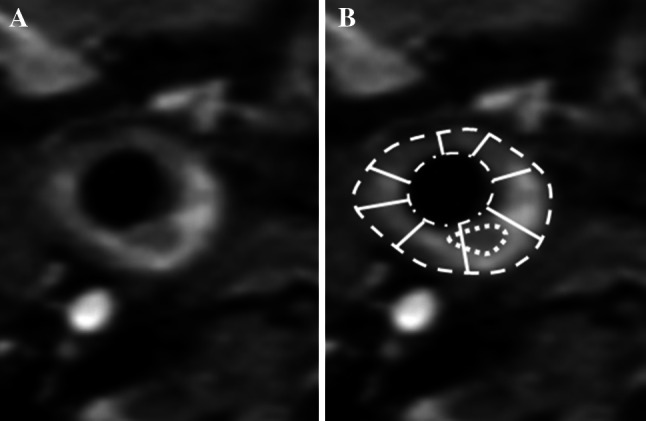
A T1-weighted contrast-enhanced fat saturation image through the common carotid depicts the vessel morphology including the lumen area (*dot-dash line*), total vessel area (*dashed line*) and mean wall thickness (a value obtained by averaging a number of cords, represented by the solid lines). The wall area is calculated by subtracting the lumen area from the total vessel area. The lipid-rich necrotic core component is also outlined (*dotted line*)

Quantitative information can be abstracted from imaging data through vessel wall segmentation. Performing this task manually is labor-intensive and subject to inter- and intra-observer variability. Post-processing software allows for semi- and fully automated multi-planar assessment of plaques for both qualitative and quantitative analysis. Various methods have been tried including image deformation [46], region growing algorithms [47] and model-based segmentation [48], to name a few. These computer-aided techniques are used to assess different measures of carotid morphology including the lumen area, total vessel area (sometimes called the outer wall area), wall area and mean wall thickness (Fig. 5). These methods help ensure that the inter-scan reproducibility of both vessel morphology and tissue composition measurements, such as the volume of lipid-rich necrotic core and calcification, is high, and the intraclass correlation for these techniques is large, with coefficients ranging from 0.87 to 0.99 [49]. Thus facilitated by computation support, MRI provides a reliable tool for longitudinal carotid assessment [50].

## Clinical outcomes

The presence of these complex plaque components correlates with traditional cardiovascular risk factors [51–53]. For instance, Wasserman et al. [54], demonstrated that in asymptomatic individuals with thickened carotid walls, the presence of lipid core by MRI is associated with total plasma cholesterol. Features such as a thin fibrous cap, large fatty or necrotic core and intraplaque hemorrhage are associated with plaque instability [55]. Intraplaque hemorrhage is a feature of complicated late-staged atherosclerotic plaque (see Fig. 4), thought to be the result of leaky neo-capillaries [56] and associated with sustained acceleration of plaque progression [57]. Complex morphology, including plaque ulceration [58], and these unstable plaque components, predict a higher likelihood of plaque rupture, resulting in thromboembolism that culminates in stroke [59–66].

As a surrogate marker of disease within other vascular beds, carotid atherosclerosis has been shown to predict the presence of coronary artery disease and its manifestations such as angina, myocardial infarct, resuscitated cardiac arrest and coronary atherosclerosis related death [7, 67].

## Future applications

As discussed, there is ample evidence of the prognostic value of MRI in the prediction of future stroke and preliminary data regarding the value of this imaging technique in the prediction of coronary events. Further research is still needed to determine if measured changes in plaque volume and imaging characteristics connote a similar reduction in future cerebrovascular, and possibly even cardiovascular, risk. Despite the robust performance of carotid MRI as a prognostic marker, its potential for widespread clinical adaption will likely be heavily influenced by its availability and cost-effectiveness.

## Conclusion

The carotid artery is a high-yield target for cardiovascular risk. The technical advantages provided by carotid MRI allows for the characterization of unstable plaque components. Not only does MRI imaging of carotid atherosclerosis predict stroke, but atherosclerosis in the carotid arteries is also indicative of cardiac outcomes, providing a mechanisms with which to more thoroughly screen patient groups. Non-invasive imaging techniques for vascular assessment have the potential to provide biomarkers for use in future research studies.
